# Roles of differential expression of miR-543-5p in GH regulation in rat anterior pituitary cells and GH3 cells

**DOI:** 10.1371/journal.pone.0222340

**Published:** 2019-09-11

**Authors:** Ze-Wen Yu, Wei Gao, Xin-Yao Feng, Jin-Yu Zhang, Hai-Xiang Guo, Chang-Jiang Wang, Jian Chen, Jin-Ping Hu, Wen-Zhi Ren, Bao Yuan

**Affiliations:** Department of Laboratory Animals, College of Animal Sciences, Jilin University, Changchun, Jilin, P.R. China; Nanjing Agricultural University, CHINA

## Abstract

Growth hormone (GH) is an important hormone released by the pituitary gland that plays a key role in the growth and development of organisms. In our study, TargetScan analysis and the dual luciferase reporter assays were used to predict and screen for miRNAs that might act on the rat *Gh1* gene, and we identified miR-543-5p. Then, the GH3 cell line and the primary rat pituitary cells were transfected with miRNA mimic, inhibitor, and siRNA. We detected the *Gh1* gene expression and the GH secretion by real-time PCR and ELISAs, respectively, to verify the regulatory effect of miR-543-5p on GH secretion. The results showed that miR-543-5p can inhibit *Gh1* mRNA expression and reduce GH secretion. MiR-543-5p inhibitor upregulated *Gh1* mRNA expression and increased GH secretion compared with the negative control. In summary, miR-543-5p downregulates *Gh1* expression, resulting in a decrease in GH synthesis and secretion, which demonstrates the important role of miRNAs in regulating GH and animal growth and development.

## Introduction

As a major hormone in the rat pituitary gland, growth hormone (GH) plays an important role in regulating the growth and metabolism of organisms[1–3], and GH production is controlled by central and peripheral signals[4]. The hypothalamus also plays a key role in the production and release of GH and regulates GH secretion through GH releasing factors or GH release-inhibiting factors[5]. GH not only promotes bone proliferation but is also indispensable in the differentiation of chondrocytes.[6] Excessive GH can cause cell aging and may also result in certain cancers[7–9]. Therefore, it is necessary to control the time and extent of its effect to prevent GH from triggering physiological disorders in organisms[10]. GH primarily acts on the liver. Successful expression of hepatic insulin-like growth factor 1 (*Igf-1*) mRNA is also associated with GH[11–13]. To some extent, the activity of the GH/IGF-1 axis is considered to be related to age. The finding that individuals over 60 years old have particularly low levels of GH showed that as age increases, endocrine and paracrine *Igf-1* provides decreased nutrient levels to support the brain[14, 15]. Therefore, it is necessary to further elucidate the factors that affect GH secretion.

MicroRNAs (miRNAs) are a class of non-coding RNAs ranging from 18 to 25 nucleotides in length that are widely found in single-cell eukaryotes, animal cells, and plant cells[16]. In organisms, miRNAs generally control gene expression (usually inhibition of target gene expression) by targeted recognition of the 3' untranslated region (UTR) of the mRNA[17]. MiRNAs can also modify histones or methylation of promoter sites in certain cases, which may facilitate or inhibit gene[18]. Multiple studies have shown that miRNAs can regulate hormone expression. For example, miR-136-3p inhibited the expression of *Lhr*[19], while the synthesis of FSH and LH was promoted by miR-7a2[20]. However, the effects of miRNAs on GH secretion still need to be studied.

The pituitary gland is a small endocrine gland in the brain that is located at the sphenoid bone depression in the lower hypothalamus, which is connected to the pituitary stalk. The hypophyseal gland includes the pituitary gland and the neurohypophysis gland[21]. The pituitary gland, a small organ, can release hormones, which all play an essential role in biological activities[22, 23]. Thus, in-depth studies of pituitary miRNAs are needed to obtain a deeper understanding of the effects of miRNAs on the development of organs, individual animals and species characteristics.

In this work, we identified the interaction between miRNA-543-5p and the *Gh1* 3'UTR using a dual luciferase reporter and transfected miRNA-543-5p mimic and inhibitor in a GH3 cell line and rat pituitary primary cells. We measured the expression of *Gh1* miRNA and the secretion of GH and demonstrated that miRNA-543-5p affects GH secretion.

## Materials and methods

### Ethics statement

All experiments were performed in accordance with the relevant guidelines of the Guide for the Care and Use of Laboratory Animals of Jilin University. All animal procedures were conducted under the protocol approved by the Institutional Animal Care and Use Committee (IACUC) of Jilin University (201805027).

### Animals and cell culture

7-day-old, 40-day-old, 90-day-old, 250-day-old and four-month-old healthy male Sprague-Dawley rats were obtained from Liaoning Changsheng Biotechnology, Co. Ltd. We used the rats to determine whether miR-543-5p affected GH expression. We euthanized Four-month-old rats after carbon dioxide anesthesia and then carried out cervical dislocation. The heads were removed. Then, we sheared the skin along the two lines between the ears and mouth to expose the skull. Two symmetrical white lines on the rat skull were clearly observed. We sheared along the two lines, opened the skull with tweezers, removed the pituitary glands and placed them into precooled PBS supplemented with 0.3% BSA (Sigma, USA) and 1% penicillin/streptomycin (HyClone, USA), and the blood was washed from the pituitary. Then, we separated the neurohypophysis from the pituitary and used ophthalmic scissors to cut the tissue into pieces in Dulbecco’s modified Eagle’s medium/Nutrient Mixture F12 (DMEM/F12) (HyClone, USA), containing 2.5% collagenase type I (Gibco, USA). After a 90 min incubation with atmosphere 5% CO_2_ at a temperature of 37°C, we diluted the cell suspension with PBS (0.3% BSA and 0.1% penicillin/streptomycin). Then, we filtered the cell suspension with a 200 mesh (75 μm) cell sieve to remove the cell clusters and undigested tissue. Next, the cell suspension was centrifuged at 200 g for 10 min. Finally, 2 mL of the DMEM/F12 culture medium with 20% fetal bovine serum (FBS) was used to resuspend cell pellet. The cells were cultured in a 37°C incubator with 5% CO_2_.

The culture medium was replaced with fresh medium at 48 h to maintain the cells. All experiments were carried out under sterile conditions. The GH3 cell line was passaged three or four times a week. The passage ratio was 1:2~1:4. Before transfection, we transferred cells from culture bottles to 24-well plates.

### SiRNA,MiRNA mimic and inhibitor transfection

We seeded GH3 cells and rat anterior pituitary cells, and after they were adherent and 50% confluent, we transfected siRNA(GenePharma), mimic negative control (nc), mimic nc with fluorescent markers, mimic, inhibitor negative control (i-nc) and inhibitor with the riboFECT CP Transfection Kit (RiboBio Biotech, Guangzhou, China)(mimic negative control: UUUGUACUACACAAAAGUACUG; inhibitor negative control: mCmAmGmUmAmCmUmUmUmUmGmUmGmUmAmGmUmAmCmAmAmA; miR-543-5p mimic: AAGUUGCCCGCGUGUUUUUCG; miR-543-5p inhibitor: mCmGmAmAmAmAmAmCmAmCmGmCmGmGmGmCmAmAmCmUmU; m refers to 2'-Ome); the concentrations were all 100 nM. After 48 h of incubation, all cells were used for the following experiments.

### RNA isolation and quantitative real-time PCR detection

We extracted total RNA from harvested cells by TRIzol reagent (Tiangen, Beijing, China) strictly following the manufacturer’s protocol and then used the NanoDrop ND-2000 spectrophotometer to measure the consistency and OD value to ensure the RNA quality. According to the concentration we measured, we obtained cDNA through the FastQuant RT Kit (with gDNase) following the manufacturer’s instructions (Tiangen, China). Then, we performed a q-PCR reaction with a 20 μL volume on a Mastercycler ep realplex^2^ system (Eppendorf, Germany). The q-PCR reaction mix consisted of 1 μL cDNA, 10 μL of 2×SuperReal PreMix Plus (Tiangen), and 0.5 μL of each primer, which was 10 μM. Water was added to a volume of 20 μL. The mRNA and miRNA primers are listed in the S1 File.

### Construction of the reporter plasmids

Based on the rat *Gh1* mRNA sequence in TargetScan, we designed primers and amplified the 3'UTR sequences of rat *Gh1* mRNA by PCR. The PCR product was cloned between the XhoI and NotI sites of the pmiR-RB-REPORTTM plasmid, generating the pmiR- *Gh1*-3'UTR-WT plasmid (S2 File). Then, a double-stranded oligonucleotide with site-specific mutations in the miR-543-5p sequences was also cloned into the same plasmid, producing pmiR- *Gh1*-3'UTR-MUT (S3 File) (Guangzhou RiboBio Biotech Co., Ltd.), for construction of the reporter plasmids. All PCR products were verified by DNA sequencing (RiboBio Biotech Co., Ltd., Guangzhou, China).

### Dual-luciferase reporter assay

We added 1.5×10^4^ 293T cells in each well of a 96-well plate and seeded the cells for 24 h until transfection. The next day, 50 ng of reporter plasmid along with the miRNA mimic or nc at a concentration of 50 nM was cotransfected using Lipofectamine^™^ 3000 (Invitrogen, USA). The fluorescence intensity meter (Veritas 9100–002) was used to measure fluorescence values after 48 h of transfection.

### Detection of cell apoptosis through flow cytometry

We used the Annexin V-FITC/PI Apoptosis Kit to detect the apoptosis of rat anterior adenohypophysis cells and GH3 cells (Multi Sciences, Hangzhou, China). Then, we used trypsin to digest adherent cells after transfection for 24 h. For the GH3 cell line, cells were centrifuged at 1000× rpm for 5 min and harvested by cell sedimentation. For anterior pituitary cells, cells were centrifuged at 200×g for 5 min and harvested by cell sedimentation. In addition, the 10× Binding Buffer was diluted in distilled water to obtain a 1× Buffer, which was added into each 1.5 mL centrifuge tube at 100 μL. We used four groups: cells without treatment that were dyed with PI, FITC or both PI and FITC, and the other group consisted of the sample group dyed with both PI and FITC. We added V-FITC (5 μL) into the Annexin V-FITC single-dye tube and PI (10 μL) into the PI single-dye tube, and sample tubes and the double-dye tubes were supplemented with both 5 μL of Annexin V-FITC and 10 μL of PI. Finally, we analyzed cell apoptosis via flow cytometry at 2 h.

### GH secretion detection

We collected 50 μL of the culture medium and GH3 cells after transfection of mimics, mimic nc inhibitor and i-nc for 24 h. Then, the Rat GH ELISA Kit (Haling Biotech Co., Ltd., Shanghai, China) was used to measure the secretion of GH in the culture medium. For anterior pituitary cells, we also collected 50 μL the culture medium and rat anterior pituitary cells for ELISAs after 24 h of transfection.

### Statistical analysis

All the data were obtained from three independent experiments and are expressed as the mean±standard deviation. One-way ANOVA and independent-samples t tests were performed to evaluate the statistical significance of differences. P<0.05 was considered statistically significant.

## Results

### MiR-543-5p targets the 3'UTR of *Gh1* mRNA

**Fig 1:**
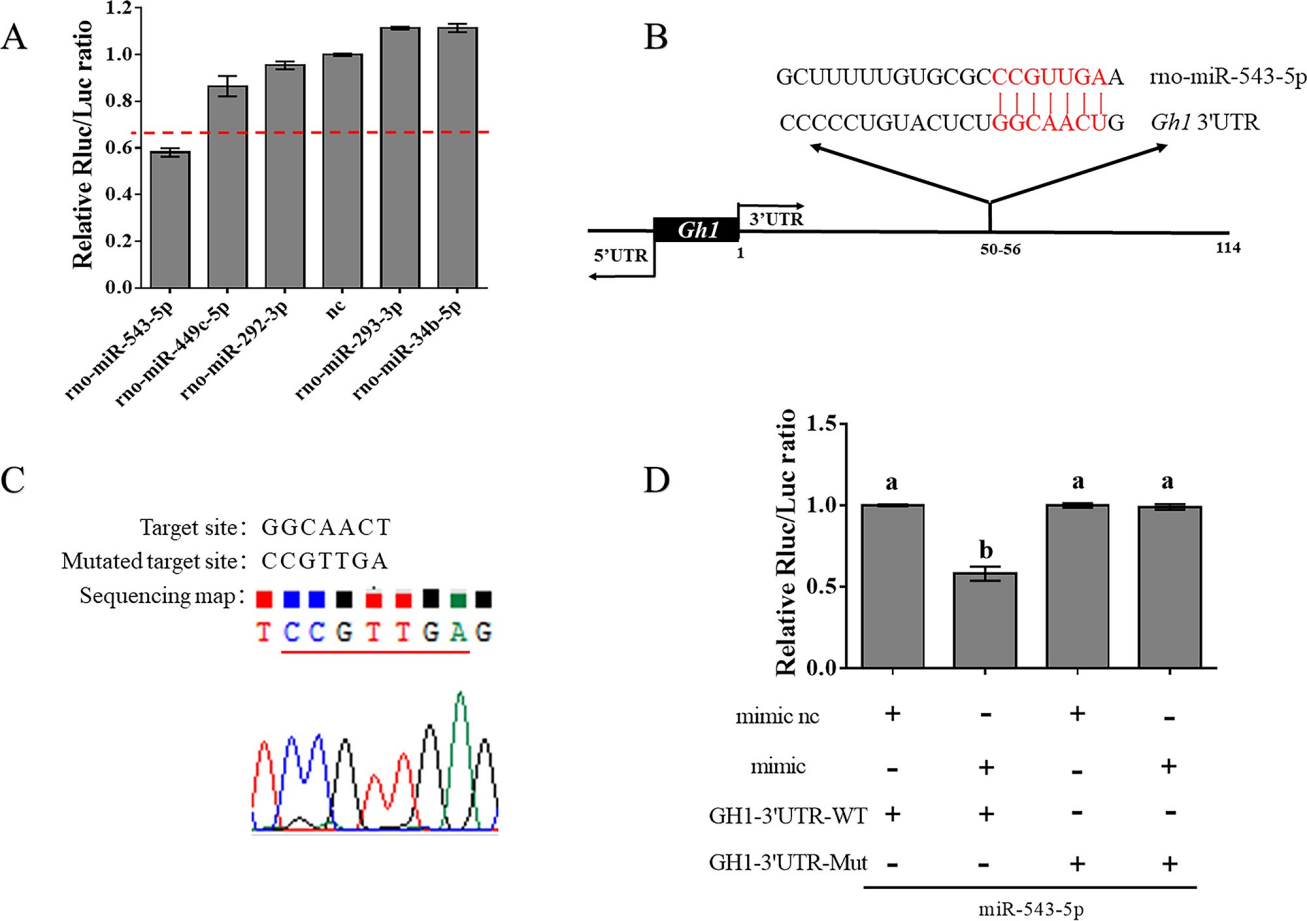
MiR-543-5p targets the 3’ UTR of *Gh1* mRNA. (A) Identification of miRNAs which may target the *Gh1* 3’UTR. Changes of relative luciferase activity of the pmiR- *Gh1*-3’UTR-WT vector after the 5 candidate miRNAs were transfected into 293T cells. Measuring the relative luciferase activity when the time of transfection reach 48 hours. The normalized relative Rluc/Luc activity ratio for the negative control was set to 1. (B) The base complementary pairing sequence of miR-543-5p and the *Gh1* 3′ UTR predicted by TargetScan is marked in red. (C) The target site was mutated from GGCAACT to CCGTTGA. The sequencing map showed the sequence. (D) Examining the relative luciferace activity after co-transfection of the plasmid with the miR-543-5p nc/mimic. The luciferase activity of co-transfection of *Gh1*-3’UTR WT plasmid and nc group was set to 1 as the negative control. Each experiment was tested over three times. Data are shown as means ± SD. One-way ANOVA was used to assess statistical significance, Different letters a and b exhibit differences significant between groups (p< 0.05).

First, we used TargetScan to predict the miRNAs that could potentially target the 3'UTR of the rat *Gh1* gene. Then, we identified 5 candidate miRNAs, including miR-293-3p, miR-292-3p, miR-543-5p, miR-449c-3p, and miR-34b-3p (S1 Table). These miRNAs may target the 3'UTR of *Gh1* mRNA and affect *Gh1* mRNA expression post-transcriptionally. Next, we constructed a reporter plasmid, pmiR- *Gh1*-3'UTR-WT, to identify which miRNAs can lead to a more than 30% reduction in luciferase activity. We cotransfected the pmiR- *Gh1*-3'UTR-WT plasmid and miR-543-5p mimic into 293T cells and found that the luciferase activity was reduced 42% (Fig 1A). Then, according to the base complementary region information from TargetScan (Fig 1B), we mutated the target complementary sequence GGCAACT to CCGTTGA to construct the reporter plasmid pmiR- *Gh1*-3'UTR-MUT (Fig 1C). The mutated plasmid was also cotransfected with miR-543-5p mimic. However, we found that the cotransfection of miR-543-5p and pmiR- *Gh1*-3'UTR-MUT yielded no changes in luciferase activity (Fig 1D). As a result, we selected miR-543-5p, which could directly target *Gh1* to regulate its expression.

### The expression levels of miR-543-5p in different developmental stages and in various rat tissues

**Fig 2:**
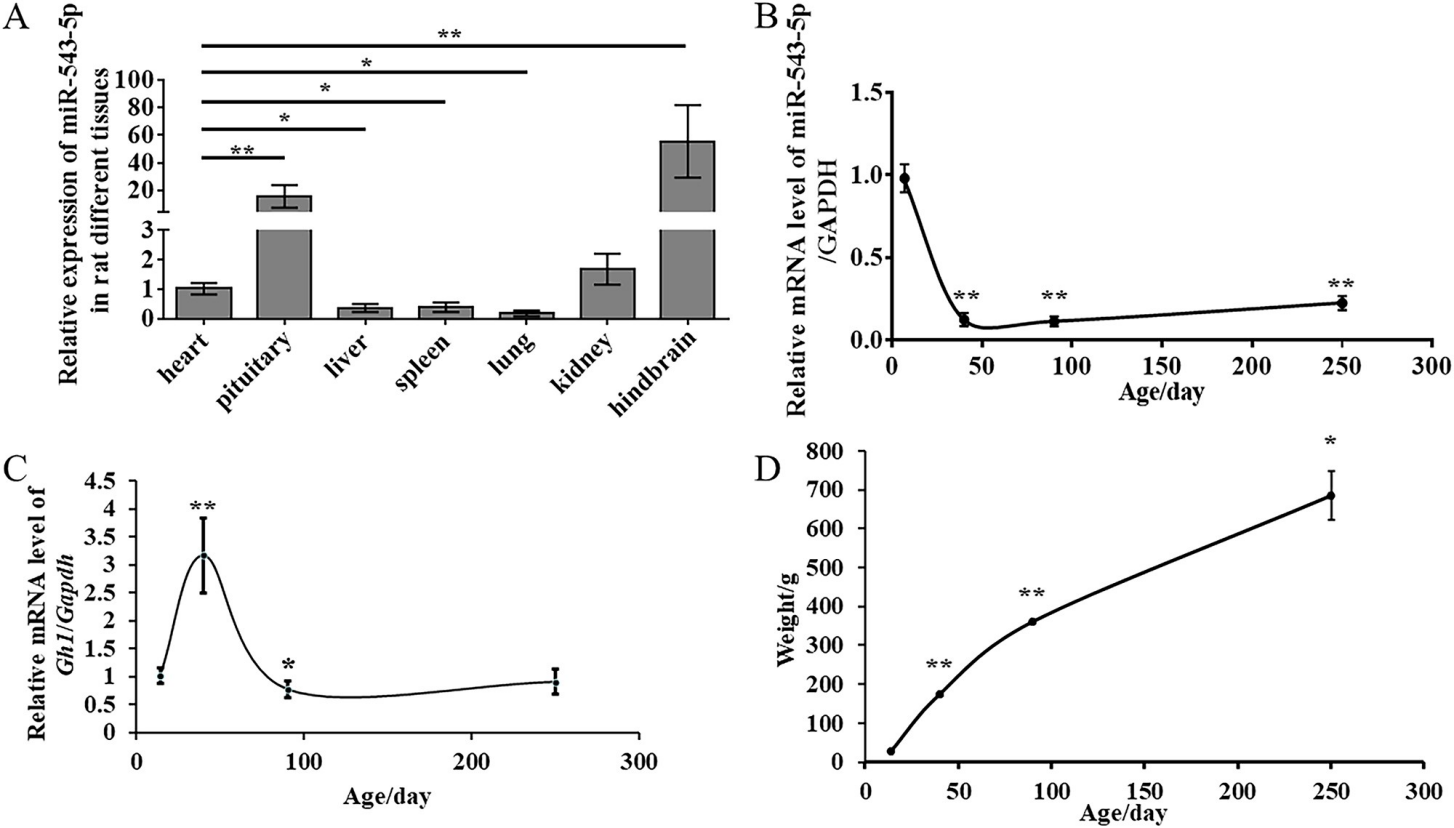
The expression level of miR-543-5p in different development stages and in various rat tissues. (A) Analysis of miR-543-5p levels detected by q-PCR in rat different tissues. (B) Analysis of miR-543-5p levels detected by q-PCR detected in two-week-old rats and four-month-old rats. (C) Analysis of *Gh1* levels detected by q-PCR detected in 7-day-old, 40-day-old, 90-day-old and 250-day-old rats. (D) Growth curve of the body weight in different ages rats with 7-day-old, 40-day-old, 90-day-old and 250-day-old. Each experiment was tested over three times. Data are shown as means ± SD. One-way ANOVA and independent-samples t test were used to analyse statistical significance (*P<0.05; **P<0.01).

First, we dissected sexually mature rats, collected seven tissues from the rats, and detected the miR-543-5p expression patterns. We found that miR-543-5p was barely expressed in heart, liver, spleen, lung and kidney but was highly expressed in the hindbrain (55-fold) and relatively highly expressed in the pituitary gland (16-fold) (Fig 2A). Then, we selected 7-day-old rats as the non-sexually mature group, 40-day-old rats as the sexually mature group, 90-day-old rats as the physically mature group and 250-day-old rats as the stable weight group, extracted their anterior pituitaries and detected the expression levels of miR-543-5p. The results showed that the miR-543-5p expression in the pituitary gland of the sexually mature group was the lowest among all the groups (Fig 2B). We also detected the expression pattern of *Gh1* during the developmental stages of rat pituitary using 7-day-old, 40-day-old, 90-day-old, 250-day-old rats (Fig 2C), and the relevant growth curve of the body weight of rats of different ages was measured (Fig 2D).

These results indicated that rat body weight, pituitary *Gh1* and pituitary miR-543-5p were all markedly age-dependent. The level of pituitary miR-543-5p was the highest on Day 7 and declined rapidly until Day 40 and increased gradually with age. The level of pituitary *Gh1* increased rapidly after Day 7, reached a peak on Day 40 and then declined until the expression level had near level no change. Body weight was the lightest on Day 7 and kept increasing with age after birth. When the level of *Gh1* high expresses, the level of miR-534-5p expresses low. That means miR-543-5p affect the expression of *Gh1*.

### The effect of transfection of miR-543-5p on the GH3 cell line and rat pituitary cells

**Fig 3:**
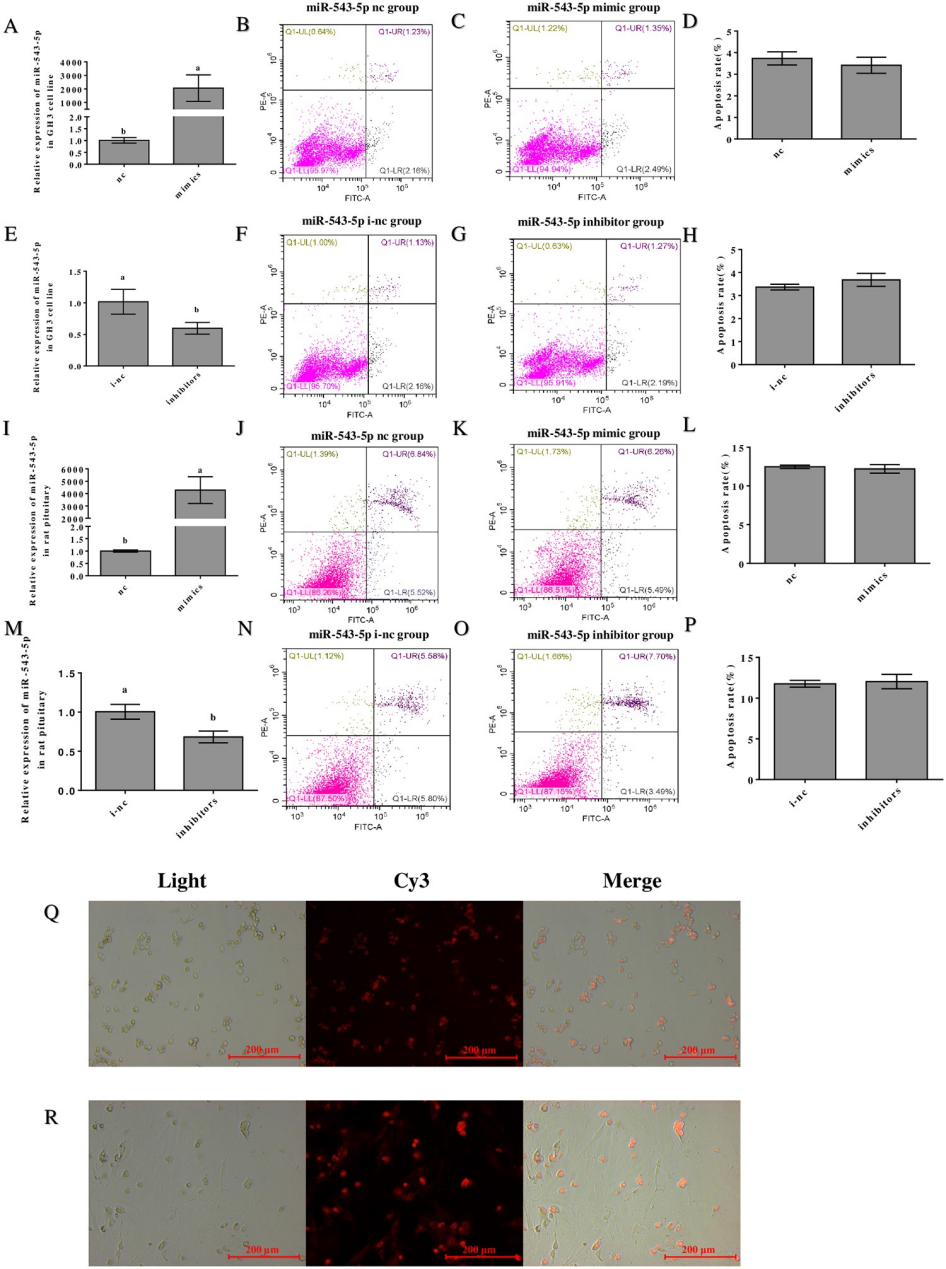
The effect of transfection of miR-543-5p on GH3 cell line and rat pituitary cells. (A/E) Analysis of miR-543-5p levels detected by q-PCR after transfection with miR-543a-5p mimic/inhibitor in GH3 cells. (I/M) Analysis of miR-543-5p levels detected by q-PCR after transfection with miR-543a-5p mimic/inhibitor in rat anterior pituitary cells. Observe apoptosis by flow cytometry after staining GH3 cells and rat anterior pituitary cells which were transfected. (B) miR-543-5p nc group in GH3 cell line. (C) miR-543-5p mimic group in GH3 cell line. (F) miR-543-5p i-nc group in GH3 cell line. (G) miR-543-5p inhibitor group in GH3 cell line. (D/H) The percentage of apoptotic GH3 cells after transfection with miR-543-5p nc/mimic/ i-nc/ inhibitor. (J) miR-543-5p nc group in rat anterior pituitary cells. (K) miR-543-5p mimic group in rat anterior pituitary cells. (N) miR-543-5p i-nc group in rat anterior pituitary cells. (O) miR-543-5p inhibitor group in rat anterior pituitary cells. (L/P) The percentage of apoptotic rat anterior pituitary cells after transfection with miR-543-5p nc/mimic/ i-nc/ inhibitor. Fluorescence labeling was detected after transfection with the nc mimic carrying fluorescence markers in GH3 cells (Q) and rat anterior pituitary cells (R). Each experiment was tested over three times. Data are shown as means ± SD. Independent-samples t test was used to analyse statistical significance, Different letters a and b exhibit differences significant between groups (p< 0.05).

To demonstrate the transfection effect of miR-543-5p mimic nc, mimic, inhibitor nc and inhibitor, we used q-PCR to detect the expression levels of miR-543-5p in GH3 cells and rat pituitary cells after transfection. For GH3 cells, transfection of mimic resulted in an obvious increase in the expression level of miR-543-5p. Conversely, the expression level of miR-543-5p showed a significant decrease following transfection of inhibitor (Fig 3A and 3E). The same results were observed in rat anterior pituitary cells (Fig 3I and 3M). In addition, to determine the transfection efficiency, we transfected GH3 cells and rat anterior pituitary cells with mimic nc with fluorescent markers and detected red fluorescence labeling in the cells, revealing that the transfection was successful (Fig 3Q and 3R). For quality control, we detected the apoptosis of different cell lines after transfection by flow cytometry. First, miR-543-5p mimic nc, mimic, inhibitor nc, and inhibitor were transfected in GH3 cells for 24 h. The cell apoptosis results showed no significant difference between the negative control group and the transfection group (Fig 3B–3D and 3F–3H). Similarly, we also detected apoptosis of rat pituitary cells transfected with miR-543-5p mimic nc, mimic, inhibitor nc, and inhibitors for 24 h, and the results were the same as those of the transfection experiments in GH3 cells (Fig 3J–3L and 3N–3P). The results clearly indicate that our transfection experiment is accurate and reliable.

### The effects of overexpressing or inhibiting miR-543-5p on the expression level of *Gh1* and secretion level of GH in GH3 cells and in rat anterior pituitary

**Fig 4:**
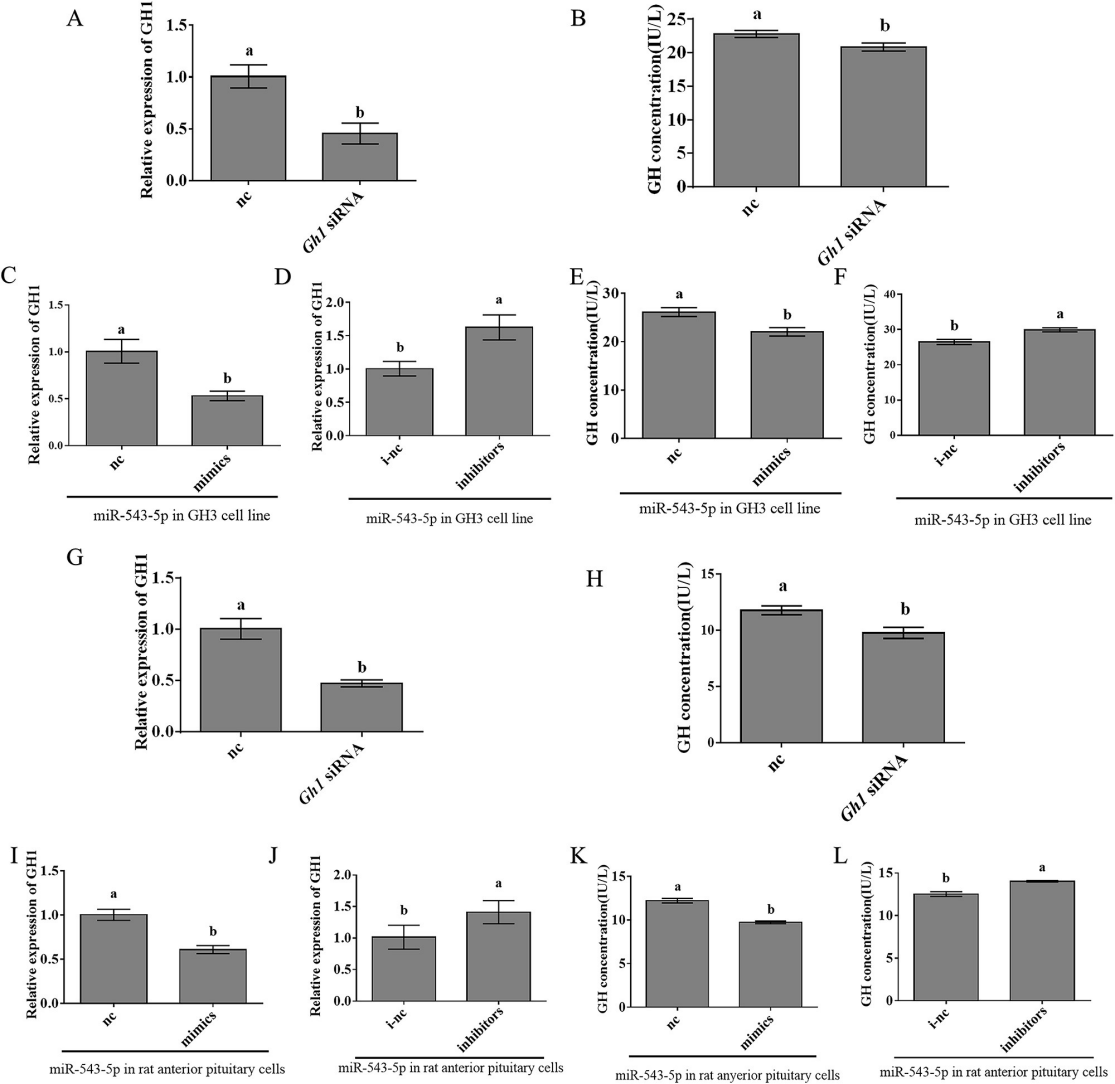
The effects of overexpressing or inhibiting miR-543-5p on the expression level of *Gh1* and the secretion level of GH in GH3 cell line and in rat anterior pituitary. *Gh1* relative expression (A) and GH concentration (B) after transfection with siRNA in GH3 cell line. (C-D) Analysis of *Gh1* levels detected by q-PCR in miR-543-5p nc, mimic, i-nc, and inhibitor groups in GH3 cell line. (E-F) Analysis of GH concentration detected by ELSIA in miR-543-5p nc, mimic, i-nc, and inhibitor groups in GH3 cell line. *Gh1* relative expression (G) and GH concentration (H) after transfection with siRNA in rat anterior pituitary cells. (I-J) Analysis of Gh1 levels detected by q-PCR in miR-543-5p nc, mimic, i-nc, and inhibitor groups in rat anterior pituitary cells. (K-L) Analysis of GH concentration detected by ELSIA in miR-543-5p nc, mimic, i-nc, and inhibitor groups in rat anterior pituitary cells. Each experiment was tested over three times. Data are shown as means ± SD. Independent-samples t test was used to analyse statistical significance, Different letters a and b exhibit differences significant between groups (p< 0.05).

Transfection experiments were performed to verify the interaction of miR-543-5p with *Gh1* in rat pituitary. First, as positive controls, *Gh1* siRNA was transfected into the GH3 cell line and rat pituitary primary cells, and we detected *Gh1* mRNA expression level and GH secretion level by Q-PCR and ELISAs after transfection for 24 h. As expected, the *Gh1* mRNA level and GH secretion decreased significantly after transfection with *Gh1* siRNA (Fig 4A, 4B, 4E and 4F). Then, we transfected miR-543-5p mimic nc, mimic, inhibitor nc, and inhibitor into GH3 cells and rat pituitary cells and detected *Gh1* mRNA expression level and GH secretion level after 24 h. In the GH3 cell line, *Gh1* mRNA expression was significantly decreased after overexpressing miR-543-5p compared with that of the nc group. In contrast, the mRNA expression of *Gh1* was significantly increased after inhibiting miR-543-5p (Fig 4C). In rat anterior pituitary cells compared with the nc group cells, the expression level of *Gh1* mRNA was significantly decreased after overexpression of miR-543-5p, and the mRNA expression level of *Gh1* was also significantly increased after inhibition of miR-543-5p (Fig 4E). The GH secretion level was detected in the GH3 cell line and rat pituitary cells after transfection for 24 h. The GH secretion level had the same trend as *Gh1* expression (Fig 4D and 4H). These results demonstrated that miR-543-5p can inhibit *Gh1* expression and reduce GH secretion.

## Discussion

Analysis of changes in fluorescence activity after cotransfecting mimic and constructed plasmid or mutant plasmid can be used to determine the interaction between candidate miRNAs and target genes[24, 25]. In our study, we cotransfected miR-543-5p mimic and pmiR- *Gh1*-3'UTR-WT into 293T cells and found that the luciferase activity was reduced by over 40%. After the target site was mutated, luciferase activity was significantly corrected. Therefore, we proved that miR-543-5p has a regulatory effect on *Gh1* gene expression.

To date, miRNAs have been found to participate in many biological systems. For example, they play roles in stem cell differentiation[26]. the immune system[27]. lipid metabolism[28], growth and development[29], In addition, approximately 68% of miRNAs are highly tissue-specific[30]. The pituitary gland is an important endocrine organ in organisms. Increasing evidence has shown that miRNAs play a vital role in both pituitary tumors and normal pituitary. For example, in pituitary adenomas, dysregulation of miRNAs may lead to cell cycle disorders[31]. The miR-148b and miR-152/alcam axes have inhibitory effects on cell proliferation and invasion[32]. MiR-21 can inhibit the expression of PITX 2 to promote apoptosis[33]. All these findings reveal that miRNAs affect the cell cycle, cell proliferation, cell invasion, apoptosis and other processes. In the normal pituitary, miRNAs may regulate the postnatal growth of pigs[34]. The development of the pituitary gland in mouse is also regulated by miR-26b[35]. In this study, transfection of miR-543-5p related molecules did not promote apoptosis in rat anterior pituitary cells and GH3 cell lines.

According to previous studies, miR-543-5p has been found in a variety of cancers and has unique pathological significance. MiR-543-5p was shown to be involved in colorectal cancer[36], gastric cancer[37], breast cancer[38] and osteosarcoma cells[39]. In addition, miR-543 can also be involved in the regulation of the biological processes of normal cells. MiR-543-5p regulates the senescence of human bone marrow mesenchymal stem cells by decreasing the expression level of AIMP 3/p18[40]. In addition, miRNAs play a unique role in the secretion of various hormones in the pituitary. For example, miR-141-3p regulates the secretion of GH hormones[14]. MiR-325-3p is associated with the immobilization-induced suppression of LH secretion, and miR-375 is involved in ATCH secretion. In porcine anterior pituitary cells, miR-361-3p regulate FSH by directly interacting. MiR-122 can downregulate the expression of *Lhr* mRNA through the SREBP pathway to inhibit the secretion of LH[41], and miR-9 can promote lactation by regulating D2 receptors[42]. Moreover, our previous studies have shown that miR-21-3p, miR-433 and miR-186-5p could affect the expression of *Fshβ* mRNA. Therefore, these studies have demonstrated that miR-543-5p affects cell proliferation, senescence and apoptosis. However, the relationship between miR-543-5p and hormone secretion is still unclear. According to our experiments, overexpression of miR-543-5p can reduce the expression level of *Gh1*, thereby inhibiting GH secretion.

MiRNAs, which are widely present in organisms, play a significant role in various tissues and organs and exhibit high tissue specificity in the later stages of animal development[30]. According to previous studies, a variety of miRNAs expressing tissue specificity were identified in the pig genome[43]. In addition, miR-1 has been found to be a highly sensitive marker for early AMI due to its muscle specificity[44]. In our study, we found that the expression levels of miR-543-5p in different tissues were different, but the expression levels in the pituitary and hindbrain were significantly higher than those of other tissues, indicating that miR-543-5p has a potential function in the pituitary gland and hindbrain.

GH is an important hormone secreted by the pituitary gland that has effects on cell growth, differentiation and metabolism[45]. Therefore, it is important to identify the relevant factors involved in GH regulation. Many factors affect the secretion of GH, and GHRH is the most important positive regulator of GH secretion[46], but little research has been done on the effect of miRNAs on GH secretion. For example, miR-26b was found to increase GH secretion by *Lef-1* in 2010[35]. In our study, we found that miR-543-5p also has regulatory effects on the *Gh1* gene. This study can help us provide a basis for GH regulation in the pituitary.

To summarize, our study demonstrated that miR-543-5p inhibits *Gh1* mRNA expression and GH secretion. This work provides a basis for the role of miRNAs in the growth and development of animals.

## Supporting information

S1 File Primers used in RT-PCRl. (PDF)

S2 File Construction of the pmiR-GH1-3'UTR-WT reporter plasmid. (PDF)

S3 File Construction of the pmiR-GH1-3'UTR-MUT reporter plasmid. (PDF)

S1 Table MiRNAs predicted by TargetScan. (PDF)
