# Supplementary material for: Roles of differential expression of miR-543-5p in GH regulation in rat anterior pituitary cells and GH3 cells
Source: PLoS One. 2019 Sep 11;14(9):e0222340. doi: 10.1371/journal.pone.0222340 (PMC6738916; doi:10.1371/journal.pone.0222340)
Supplement: S1 File — (PDF) [file pone.0222340.s001.pdf]

S1 File. Primers used in RT-PCR

**primers name**	**sequence(5'-3')**
U6 RT	CGCTTCACGAATTTGCGTGTCAT
miR-543-5p RT	CTCAACTGGTGTCGTGGAGTCGGCAATTCAGTTGAGCGAAAAAC
U6 F	GCTTCGGCAGCACATATACTAAAAT
U6 R	CGCTTCACGAATTTGCGTGTCAT
miR-543-5p F	ACACTCCAGCTGGGAAGTTGCCCGCGTGT
universal reverse	CTCAAGTGTCGTGGAGTCGGCAA
GAPDH F	GGAAACCCATCACCATCTTC
GAPDH R	GTGGTTCACACCCATCACAA
GH F	CATGCCCTTGTCCAGTCTGT
GH R	AATGTAGGCACGCTCGAACT
