# Supplementary material for: Roles of differential expression of miR-543-5p in GH regulation in rat anterior pituitary cells and GH3 cells
Source: PLoS One. 2019 Sep 11;14(9):e0222340. doi: 10.1371/journal.pone.0222340 (PMC6738916; doi:10.1371/journal.pone.0222340)
Supplement: S2 File — (PDF) [file pone.0222340.s002.pdf]

S2 File. Construction of pmiR-GH1-3’UTR-WT reporter plasmid

The full-length 3’UTR of rat GH1 mRNA (NM_001034848.2) was cloned between the XhoI and NotI sites in the pmiR-RB-REPORT^{TM} plasmid, forming the pmiR-GH1-3’UTR-WT plasmid.

The PCR colony was identified after purification of the PCR product, enzyme cleavage, purification of the cleavage product, connection and convention. The length of the product from the colony was 300 bp, similar to the theoretical length.

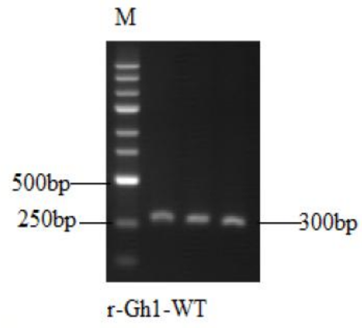

S2. Fig. 1. Results of agarose gel electrophoresis. The length of the product from the colony was 300 bp according to the markers.

The results of sequencing the positive clone were as follows (the underlined sequence is the inserted sequence, and the sequence on both sides is the vector sequence):

TGGCCTTTCGCAGGGAGGAGCTCCAGATGAATGGGTAAGTACATCAAGAGCTTCG
TGGAGCGCGTGCTGAAGAACGAGCAGTAATTCTAGGCGATCGCTCGAGCACACAC
TGGTGTCTCTGCGGCACTCCCCCGTTACCCCCCTGTACTCTGGCAACTGCCACCCC
TACACTTTGTCCTAATAAAATTAAGATGCATCATAGCGGCCGCTGGCCGCAATAAAA
TATCTTTATTTTCATTACATCTGTGTGTTGGTTTTTTGTGTGAGGATCTAAATGAGTC
TTCGGACCTCGCGGGGGCCGCTTAAGCGGTGGTTAGGGTTTGTCTGA

The length of the rat GH1 gene was 826 bp, and the full sequence (NM_001034848.2) was as follows:

CAGCACCCTCGAGCCCAGATTCCAAACTGCTCAGGTCCTGTGGACAGATCACTGA
GTGGCGATGGCTGCAGACTCTCAGACTCCCTGGCTCCTGACCTTCAGCCTGCTCTG
CCTGCTGTGGCCTCAAGAGGCTGGTGCTTTCCCTGCCATGCCCTTGTCCAGTCTGT
TTGCCAATGCTGTGCTCCGAGCCCAGCACCTGCACCAGCTGGCTGCTGACACCTA
CAAAGAGTTCGAGCGTGCCTACATTCCCGAGGGACAGCGCTATTCCATTCAGAATG
CCCAGGCTGCGTTCTGCTTCTCAGAGACCATCCCAGCCCCCACCGGCAAGGAGGA
GGCCCAGCAGAGAACTGACATGGAATTGCTTCGCTTCTCGCTGCTGCTCATCCAGT
CATGGCTGGGGCCCGTGCAGTTTCTCAGCAGGATCTTTACCAACAGCCTGATGTTT
GGTACCTCGGACCGCGTCTATGAGAAACTGAAGGACCTGGAAGAGGGCATCCAGG
CTCTGATGCAGGAGCTGGAAGACGGCAGCCCCCGTATTGGGCAGATCCTCAAGCA
AACCTATGACAAGTTTGACGCCAACATGCGCAGCGATGACGCTCTGCTCAAAAAC
TATGGGCTGCTCTCCTGCTTCAAGAAGGACCTGCACAAGGCAGAGACCTACCTGC

GGGTCATGAAGTGTCGCCGCTTTGCGGAAAGCAGCTGTGCTTTCTAGGCACACAC
TGGTGTCTCTGCGGCACTCCCCCGTTACCCCCCTGTACTCTGGCAACTGCCACCCC
TACACTTTGTCCTAATAAAATTAAGATGCATCATAAAAAAAAAAAAAAA

When the two sequences were aligned in NCBI, 100% identity was observed (Fig. 2).

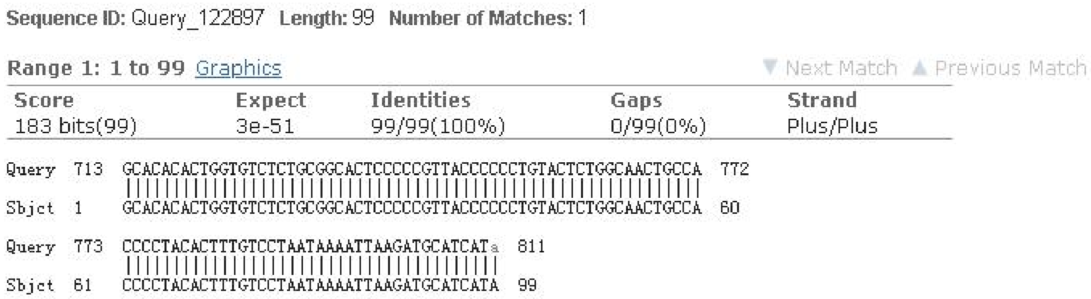

S2. Fig. 2. **Blast results.** Alignment of the inserted sequence of the positive clone with the sequence of the rat GH1 gene (NM_001034848.2). A total sequence of 99 bp was identified, which showed 100% identity.
