# Supplementary material for: Roles of differential expression of miR-543-5p in GH regulation in rat anterior pituitary cells and GH3 cells
Source: PLoS One. 2019 Sep 11;14(9):e0222340. doi: 10.1371/journal.pone.0222340 (PMC6738916; doi:10.1371/journal.pone.0222340)
Supplement: S3 File — (PDF) [file pone.0222340.s003.pdf]

S3 File. Construction of pmiR-GH1-3’UTR-MUT reporter plasmid

The full-length 3’UTR of rat GH1 mRNA was cloned between the XhoI and NotI sites of the pmiR-RB-REPORT™ plasmid. To disrupt the binding site of the GH1 3’UTR, the target sequence GGCAACT (50-56) was mutated into CCGTTGA, forming the pmiR-GH1-3’UTR-MUT plasmid.

Cloned fragment sequencing results:

r-Gh1(The full-length 3’UTR of rat GH1 mRNA)-MUT1(48-54CTGGCAA> GACCGTT)

GGCGTGCTTGCGACGTTCAGGTTCAACGTCTAATTCTAGGCGATCGCTCGAGCACACACTGGTGTCTCTGCGGCAC
TCCCCCGTTACCCCCCTGTACTGACCGTTCTGCCACCCCTACACTTTGTCCTAATAAAATTAAGATGCATCATAGC
GGCCGCTGGCCGCAATAAAATATCTTTATTTTCATTACATCTGTGTGTTGGTTTTTTGTGTGAGGATCTAAATGAG

r-Gh1(The full-length 3’UTR of rat GH1 mRNA)-MUT2(50-56GGCAACT> CCGTTGA)

CCGGCGGGGCCACGCAGGATCACGTCTAATTCTAGGCGATCGCTCGAGCACACACTGGTGTCTCTGCGGCACTCCC
CCGTTACCCCCCTGTACTCTCCGTTGAGCCACCCCTACACTTTGTCCTAATAAAATTAAGATGCATCATAGCGGCC
GCTGGCCGCAATAAAATATCTTTATTTTCATTACATCTGTGTGTTGGTTTTTTGTGTGAGGATCTAATGAGTCTTC

The plasmid was extracted from the colony, and the sequence was identified by a sequencing company. The results of sequencing showed that the target sequences were mutated successfully.

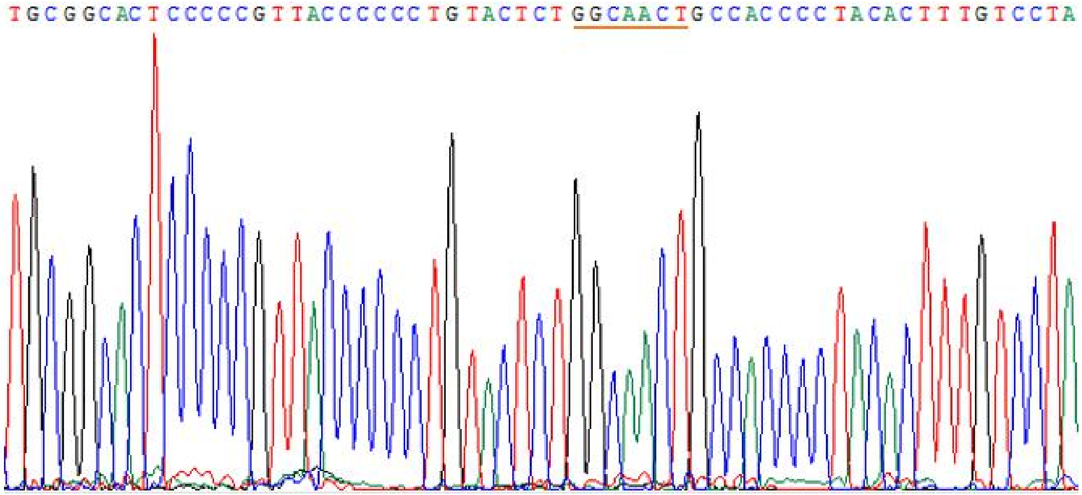

GH-WT

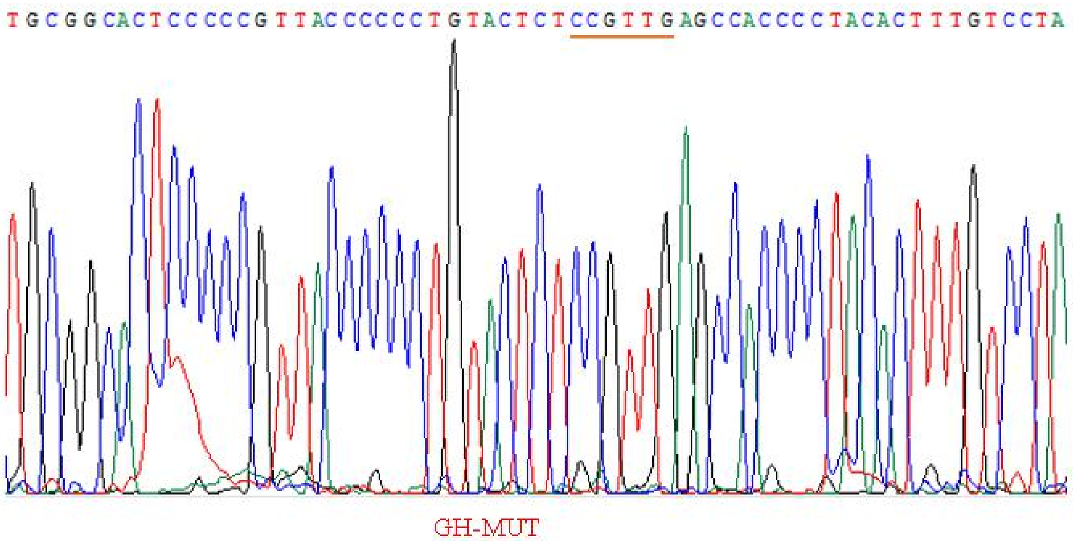

S3. Fig. 1. DNA sequence peak map. Sequence of the extracted plasmid; the target sequence GGCAACT was mutated into CCGTTGA.
