# Supplementary material for: Roles of differential expression of miR-543-5p in GH regulation in rat anterior pituitary cells and GH3 cells
Source: PLoS One. 2019 Sep 11;14(9):e0222340. doi: 10.1371/journal.pone.0222340 (PMC6738916; doi:10.1371/journal.pone.0222340)
Supplement: S1 Table — (PDF) [file pone.0222340.s004.pdf]

miRNA	Position in the UTR	seed match	context++ score	context++ score percentile	weighted context++ score	conserved branch length	Pct
rno-miR-293-3p	19-25	7mer-m8	-0.58	96	-0.58	0	N/A
rno-miR-292-3p	20-26	7mer-m8	-0.4	99	-0.4	0	N/A
rno-miR-543-5p	50-56	7mer-m8	-0.39	97	-0.39	0.104	N/A
rno-miR-449c-5p	54-60	7mer-1A	-0.3	89	-0.3	0.104	< 0.1
rno-miR-34b-5p	54-60	7mer-1A	-0.3	89	-0.3	0.104	< 0.1
